# Successful Case of Intubation

**Published:** 1889-10

**Authors:** F. H. Bartlett, J. C. Clark

**Affiliations:** Olean, N. Y.; Olean, N. Y.


### A SUCCESSFUL CASE OF INTUBATION,

*With some remarks upon After-treatment.*

REPORTED BY F. H. BARTLETT, M. D., AND J. C. CLARK, M. D., OLEAN, N. Y.

Intubation of the larynx, since operators can report their cases by hundreds, has become an operation of such common occurrence that the report of an isolated case can not be a matter of particular interest to the profession unless there are circumstances attending the case—in the way of clinical history, after-treatment, or some other facts in relation to it—which impress the operator with its peculiar importance. The object, therefore, in reporting the following successful case of intubation is more particularly to call attention to the after treatment and some of the difficulties which are frequently encountered after the introduction of the tube, and regarding which the literature on the subject furnishes comparatively little information.

F. J. C., female, aged seven years, on Monday, April 8th, complained of sore throat, which developed into what was supposed to be follicular amygdalitis. This yielded readily to the usual treatment, so that she was considered well and allowed to go out of doors on the following Saturday and Sunday. On Monday, April 13th, she again complained of sore throat. This was accompanied by slight fever and slight hoarseness. She was then given tinctura ferri chloridi. This condition remained unchanged until Tuesday night, when her symptoms became worse, the hoarseness increasing, accompanied by dyspnœa and restlessness. On Wednesday diphtheritic exudation appeared on the tonsils, her temperature then being 101-5°F., and pulse 130. She was then given bichloride of mercury, $\frac{1}{32}$ of a grain every hour, with apparently some beneficial effect, as her condition on Thursday and Friday was somewhat improved. Her pulse and temperature were nearly normal, and the exudation in the throat had commenced to disappear; the hoarseness was also less marked. Saturday morning her condition became worse. There was an increase of the stenosis of the larynx, with considerable dyspnœa, and she coughed constantly. Saturday afternoon the dyspnœa increased rapidly; she became cyanotic, and preparations were made for intubating the larynx. About 5 p. m., however, during a violent paroxysm of coughing, a bit of membrane was coughed up, which greatly relieved her breathing. She immediately fell asleep and rested quietly three hours. Sunday morning the dyspnœa again increased. She from that time coughed constantly, the stenosis rapidly increased, and the general condition became grave, she showing evidences of extreme exhaustion.

Dr. M. C. Follett, Dr. C. H. Bartlett, and Dr. W. H. Sage were called in consultation, and all hope of recovery, except by means of an operation, was abandoned. Intubation was decided upon, and was successfully performed by Dr. F. H. Bartlett, with immediate relief of the dyspnœa, the patient falling asleep and resting two hours.

As has already been said, the literature on the subject gives unsatisfactory information as regards the details of the after-treatment of intubation. This being the fact, the operator of limited experience is somewhat uncertain as to the best mode of procedure. Such proved to be our experience in this case. O'Dwyer recommends giving semi-solid food, like custards, etc., to avoid irritation caused by fluids entering the trachea through the tube. This plan was tried, but there was such a loathing by the patient of anything that approached solid foods that very little was taken. Milk and brandy in small repeated doses were given, but this excited a cough, which was so distressing that it was with great difficulty that she could be induced to take any nourishment by the mouth. Enemata of beef juice and brandy were given; but the morning following the operation found our patient in no better, and perhaps a worse condition, so far as the symptoms of exhaustion were concerned, than immediately after the operation, her pulse being then over 160.

At the suggestion of Dr. J. C. Clark, who was in attendance after the operation, the patient was kept in a recumbent position when taking nourishment, with her face turned to one side, and, instead of giving her nourishment by teaspoonful doses, she was induced to drink from a feeding cup, taking as large swallows as possible and swallowing rapidly. By adopting this method we were delighted to find that she could take two ounces of milk with two drachms of brandy at each feeding with little or no difficulty and no coughing, very little of the fluid entering the tube. As soon as the effects of the increase of nourishment and stimulants began to be felt there began a steady improvement in her condition, which continued until she had fully recovered. The dyspnœa did not return after the introduction of the tube, which kept free from mucus while it was retained, it being coughed up eighty-four hours after its introduction, there being no return of the dyspnœa after its expulsion. The expectoration become muco-purulent on the second day after the operation, and on the third day bits of membrane were coughed up. During the first week after the operation she took thirty-two ounces of Cibyl's beef juice, and two ounces of brandy every twenty-four hours. Minim doses of fluid extract of digitalis were given, more especially for its diuretic effect, and on the third day, when the expectoration became scanty and tenacious, two grains of chloride of ammonium were given every hour with satisfactory results.

The patient's improvement, although slow, was satisfactory, and at this writing, although her nervous system is considerably shattered, she is slowly recovering her usual health.

